# Evolution along the Great Rift Valley: phenotypic and genetic differentiation of East African white‐eyes (Aves, Zosteropidae)

**DOI:** 10.1002/ece3.1735

**Published:** 2015-10-12

**Authors:** Jan Christian Habel, Luca Borghesio, William D. Newmark, Julia J. Day, Luc Lens, Martin Husemann, Werner Ulrich

**Affiliations:** ^1^ Terrestrial Ecology Research GroupDepartment of Ecology and Ecosystem ManagementSchool of Life Sciences WeihenstephanTechnische Universität MünchenD‐85354FreisingGermany; ^2^ C. Re Umberto 42I‐10128TorinoItaly; ^3^ Natural History Museum of UtahUniversity of UtahSalt Lake CityUtah84108; ^4^ Department of Genetics, Evolution and EnvironmentUniversity College LondonLondonWC1E 6BTU.K; ^5^ Terrestrial Ecology UnitDepartment of BiologyGhent UniversityB‐9000GhentBelgium; ^6^ General ZoologyMartin‐Luther University Halle‐WittenbergD‐06120Halle (Saale)Germany; ^7^ Chair of Ecology and BiogeographyNicolaus Copernicus University in ToruńPl‐87‐100ToruńPoland

**Keywords:** Cloud forest, disjunction, gradient, microsatellites, morphometrics, natural selection, panmixis, polyphyletic, savannah

## Abstract

The moist and cool cloud forests of East Africa represent a network of isolated habitats that are separated by dry and warm lowland savannah, offering an opportunity to investigate how strikingly different selective regimes affect species diversification. Here, we used the passerine genus *Zosterops* (white‐eyes) from this region as our model system. Species of the genus occur in contrasting distribution settings, with geographical mountain isolation driving diversification, and savannah interconnectivity preventing differentiation. We analyze (1) patterns of phenotypic and genetic differentiation in high‐ and lowland species (different distribution settings), (2) investigate the potential effects of natural selection and temporal and spatial isolation (evolutionary drivers), and (3) critically review the taxonomy of this species complex. We found strong phenotypic and genetic differentiation among and within the three focal species, both in the highland species complex and in the lowland taxa. Altitude was a stronger predictor of phenotypic patterns than the current taxonomic classification. We found longitudinal and latitudinal phenotypic gradients for all three species. Furthermore, wing length and body weight were significantly correlated with altitude and habitat type in the highland species *Z. poliogaster*. Genetic and phenotypic divergence showed contrasting inter‐ and intraspecific structures. We suggest that the evolution of phenotypic characters is mainly driven by natural selection due to differences in the two macro‐habitats, cloud forest and savannah. In contrast, patterns of neutral genetic variation appear to be rather driven by geographical isolation of the respective mountain massifs. Populations of the *Z. poliogaster* complex, as well as *Z. senegalensis* and *Z. abyssinicus*, are not monophyletic based on microsatellite data and have higher levels of intraspecific differentiation compared to the currently accepted species.

## Introduction

Oceanic islands have been extensively used as model systems to understand genetic and morphological effects of long‐term isolation and divergent selection pressures. The strong isolation and selection in these settings have often led to population differentiation, allopatric speciation, and even species radiations documented for a variety of archipelagos and organisms (Emerson 2002; Grant and Grant 2002; Whittaker et al. 2008; Husemann et al. 2014a, 2015a). Similarly, on continents geographic isolation and specific environmental conditions within certain habitat types can lead to island‐like conditions. Such ecological islands occur for example in East Africa, where moist forests restricted to isolated mountain ranges (“sky islands”) are separated from each other by a vast “sea” of dry savannah (Warshall 1994; McCormack et al. 2009).

In both types of islands (oceanic and ecological), the level of geographic isolation, geological age, and diverging ecological conditions may lead to differentiation on inter‐ and intraspecific levels (Juan et al. 2000; Measey and Tolley 2011). The moist and cool highlands of East Africa harbor cloud forests, a habitat type that is strikingly different from the adjacent dry and warm lowland savannahs. Cloud forests occur in isolated patches between 900 and 3500 m, located within a continuous lowland matrix functioning as a strong dispersal barrier for forest‐dependent species (Chapman and Chapman 1996). On the other hand, the lowland communities experience little or no such barriers to gene flow (Habel et al. 2014). These East African mountain massifs line up along the East African Rift Valley and differ in geographical isolation, geological age, altitude, and size (White 1978). The isolation of the highland habitats underlies the diversification of many species groups and has resulted in an accumulation of endemic species, often being restricted to single mountain massifs (Lovett and Wasser 1993; Fjeldså and Lovett 1997; Lovett et al. 2000; Habel et al. 2013).

Most studies focusing on genetic differentiation of East African species have either focused on the biogeographic history based on neutral molecular markers (Burgess et al. 2007; Kahindo et al. 2007; Kebede et al. 2007; Cox et al. 2014), or on the divergence of morphological or behavioral traits of species assumed to be under environmental or sexual selection (Oatley et al. 2011, 2012; Hope et al. 2012; Husemann et al. 2014b). However, the combination of both phenotypic and genetic data can help to distinguish differentiation resulting from neutral stochastic processes caused by geographic isolation and selective processes driven by local environmental conditions (Clegg et al. 2002; McKay and Latta 2002; Leinonen et al. 2006, 2008, 2013; Sæther et al. 2007; Chenoweth and Blows 2008).

In this study, we use a combination of phenotypic and molecular markers to understand differentiation processes in the East African sky island system and use the bird genus *Zosterops*, which is well known as “great speciator”, as our focal group. This genus has undergone an extensive species radiation and comprises a large number of locally endemic taxa worldwide (Slikas et al. 2000; Warren et al. 2006; Moyle et al. 2009; Milá et al. 2010; Melo et al. 2011; Cox et al. 2014). Members of the genus are found in various environments across most of East Africa (Zimmerman et al. 1996). The montane white‐eye species complex (i.e., *Z. poliogaster*) occurs exclusively at higher elevations, which has resulted in many geographically isolated populations (Cox et al. 2014). This patchy distribution caused the formation of distinct population clusters with unique genetic, phenotypic, and behavioral traits (Moreau 1957; Borghesio and Laiolo 2004; Mulwa et al. 2007; Redman et al. 2009; Habel et al. 2013, 2014; Husemann et al. 2014b, 2015b). Conversely, the yellow white‐eye, *Z. senegalensis*, can be found in both highland and lowland habitats (Zimmerman et al. 1996), and the Abyssinian white‐eye, *Z. abyssinicus,* mostly occurs in lowland savannahs, open woodland and gardens up to 1800 meters above sea level (Zimmerman et al. 1996). Based on our data, we try to disentangle the effects of different distribution patterns and divergent ecological pressures driving phenotypic and genetic differentiation in this genus. In particular, we test the following hypotheses:

- Contrasting distribution settings (population connectivity in the lowland versus disjunction of highland populations and taxa) have led to diverging inter‐ and intraspecific signatures; and
- Morphological and molecular characters exhibit diverging differentiation patterns.

## Materials and Methods

### Study species

The classification of East African *Zosterops* has been much debated. In the study region, the genus comprises 14 morphologically distinct taxonomic groups, which have been conversely split into three (Britton 1980) to seven (Mackworth‐Praed and Grant 1960) species. The most recent classification of *Zosterops*, which we adopt, recognizes at least three species in the study area (Dickinson 2003; Clements 2007; van Balen 2008). The *Z. poliogaster* (also recently named *Z. poliogastrus* according the IOC World Bird List, http://www.worldbirdnames.org) species complex occurs exclusively in the highland forests of East Africa and is found at altitudes between 1500 and 3400 m (Fry et al. 2000). Eight subspecies, with completely allopatric ranges, have been described for this species across East Africa: *Zosterops p. poliogaster* (N and E Ethiopia), *Z. p. kaffensis* (SW Ethiopia), *Z. p. kulalensis* (Mt. Kulal in N Kenya), *Z. p. kikuyuensis* (central Kenya), *Z. p. silvanus* (Taita Hills of SW Kenya), *Z. p. mbuluensis* (S Kenya and N Tanzania), *Z. p. winifredae* (South Pare Mts. of N Tanzania), and *Z. p. eurycricotus* (N Tanzania). According to the recent IOC World Bird List (http://www.worldbirdnames.org), two of the eight subspecies of *Z. poliogaster* have been given species rank: *Z. kikuyuensis* (central Kenya) and *Z. silvanus* (Taita Hills). *Zosterops senegalensis* has a broad lowland range in sub‐Saharan Africa, but within our study area, the species is restricted to highland forests, forest edges, and afroalpine scrubland from 1100 to 3400 m (Fry et al. 2000). Three subspecies are described for our study area: *Zosterops s. jacksoni* (N Kenya to N Tanzania), *Z. s. stuhlmanni* (NW Tanzania and W Kenya), and *Z. s. stierlingi* (Usambara Mts. of Tanzania). *Zosterops abyssinicus* is a lowland species, occurring in savannahs, in woodland, and at forest edges below 1500 m, with occasional records up to 2300 m (Redman et al. 2009). It comprises two subspecies within our study area: *Zosterops a. jubaensis* (S Ethiopia to N Kenya) and *Z. a. flavilateralis* (N Kenya to N Tanzania). In our subsequent analyses, we assign individuals into the following three main groups: *Z. poliogaster*, Z*. senegalensis,* and *Z. abyssinicus*. However, we consider the complex taxonomic relationships and the potential species status of some populations (see also the Discussion in this contribution).

### Sampling

**Figure 1:**
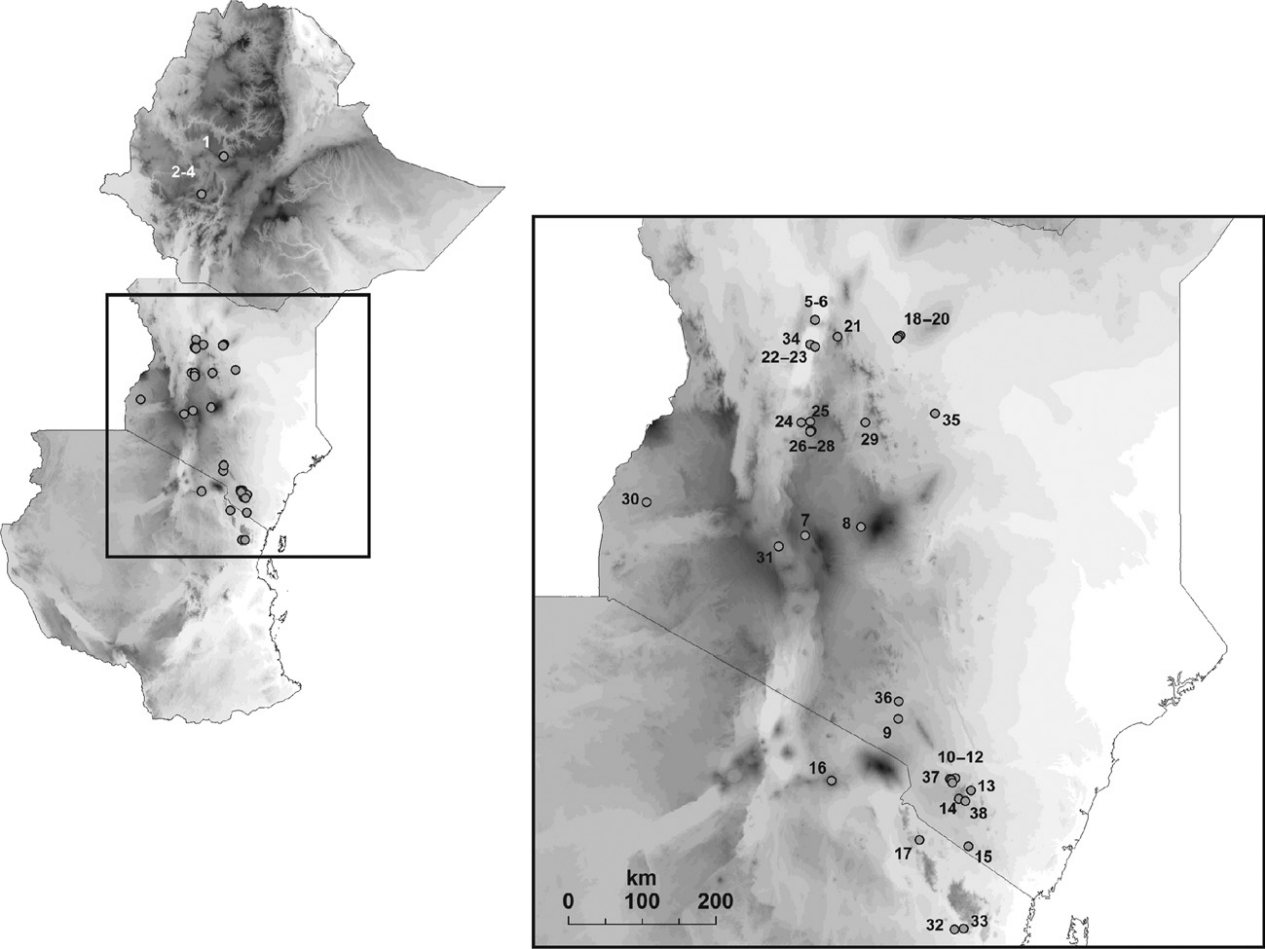
Map of all sampling locations from where morphological and molecular data were collected. Numbers of sampling locations coincide with Table 1.

We sampled populations of the three species (including most of the subspecies, further details are given in Table 1) along a latitudinal gradient in East Africa, from central Ethiopia in the north to northern Tanzania in the south. Individuals were trapped with mist nets and individually ringed. In total, five morphological characters from 1223 individuals sampled from 42 locations were measured from live birds. Feathers and blood were sampled from 385 individuals for molecular analyses for a subset of 19 locations (with 34 populations represented by morphological data, 19 populations represented by molecular data, and 14 populations for which both molecular and morphological data was available). All sampling locations are displayed in Figure 1; further details on sampling locations are provided in Table 1.

**Table 1:** Overview of the sampling locations. Given are species and subspecies, the country, region, name of location with the number of location, altitude, habitat type, and the number of individuals sampled and analyzed (phenotypic analyses *N*, molecular analyses N*) are listed. Abbreviations: E = Ethiopia, K = Kenya, T = Tanzania. Numbers of locations coincide with Figure 1

Species	Subspecies	Country/Region	Location‐Nr.	Altitude	Habitat	*N*	N*
*Zosterops poliogaster*							
*Z. poliogaster*	*poliogaster*	E‐Ethiopian Highlands	Adis Abeba‐1	2337	Cloud forest	–	12
*Z. poliogaster*	*poliogaster*	E‐Ethiopian Highlands	Jimma Hills‐2	2082	Cloud forest	–	30
*Z. poliogaster*	*poliogaster*	E‐Ethiopian Highlands	Garuke‐3	2082	Cloud forest	20	–
*Z. poliogaster*	*poliogaster*	E‐Ethiopian Highlands	Fetche‐4	1985	Cloud forest	7	–
*Z. poliogaster*	*kulalensis*	K‐Mt. Kulal	Gatab‐5	1850	Cloud forest	80	30
*Z. poliogaster*	*kulalensis*	K‐Mt. Kulal	Arabel‐6	2140	Cloud forest	5	–
*Z. poliogaster*	*kikuyuensis*	K‐Aberdares	Gatumaini Forest‐7	2342	Cloud forest	43	25
*Z. poliogaster*	*kikuyuensis*	K‐Mt. Kenya	Mt. Kenya‐8	2219	Cloud forest	30	20
*Z. poliogaster*	*mbuluensis*	K‐Chyulu Hills	Simba valley‐9	2062	Cloud forest	20	18
*Z. poliogaster*	*mbuluensis*	K‐Chyulu Hills	Satellite‐10	2200	Cloud forest	50	23
*Z. poliogaster*	*silvanus*	K‐Taita Hills	Ngangao‐11	1800	Cloud forest	42	21
*Z. poliogaster*	*silvanus*	K‐Taita Hills	Mbololo‐12	1700	Cloud forest	58	31
*Z. poliogaster*	*silvanus*	K‐Taita Hills	Chawia‐13	1600	Cloud forest	25	26
*Z. poliogaster*	*silvanus*	K‐Taita Hills	Bura‐14	1411	Cloud forest	7	7
*Z. poliogaster*	*silvanus*	K‐Mt. Kasigau	Mt. Kasigau‐15	1600	Cloud forest	42	21
*Z. poliogaster*	*eurycricotus*	T‐Mt. Meru	Mt. Meru‐16	3215	Cloud forest	–	8
*Z. poliogaster*	*winifredae*	T‐Pare Mts.	South Pare Mts.‐17	921	Cloud forest	–	8
		*N*				429	280
*Zosterops senegalensis*							
*Z. senegalensis*	*jacksoni*	K‐Mt. Marsabit	Abdul‐Ahmed camp‐18	1360	Cloud forest	147	18
*Z. senegalensis*	*jacksoni*	K‐Mt. Marsabit	Bakuli‐19	1370	Cloud forest	83	–
*Z. senegalensis*	*jacksoni*	K‐Mt. Marsabit	Lake Paradise‐20	1360	Cloud forest	67	–
*Z. senegalensis*	*jacksoni*	K‐Mt. Nyiru	Ndadapo‐21	2450	Cloud forest	14	–
*Z. senegalensis*	*jacksoni*	K‐Mt. Nyiru	Surkulé‐22	2550	Cloud forest	56	–
*Z. senegalensis*	*jacksoni*	K‐Mt. Nyiru	Chima‐23	2650	Cloud forest	3	–
*Z. senegalensis*	*jacksoni*	K‐Mt. Maralal	Ngurumaut‐24	2240	Cloud forest	33	–
*Z. senegalensis*	*jacksoni*	K‐Mt. Maralal	Sordon‐25	2450	Cloud forest	18	–
*Z. senegalensis*	*jacksoni*	K‐Mt. Maralal	Peto‐26	2070	Cloud forest	16	–
*Z. senegalensis*	*jacksoni*	K‐Mt. Maralal	Tilia‐27	2160	Cloud forest	8	–
*Z. senegalensis*	*jacksoni*	K‐Mt. Maralal	Bawa‐28	1870	Cloud forest	5	–
*Z. senegalensis*	*jacksoni*	K‐Mathews Range	Londadapo/Orokaela‐29	1870	Cloud forest	11	–
*Z. senegalensis*	*stuhlmanni*	K‐Kakamega Forest	Kakamega Forest‐30	1570	Cloud forest	11	11
*Z. senegalensis*	*jacksoni*	K‐Mau Escarpment	Eburu Forest‐31	2550	Cloud forest	6	7
*Z. senegalensis*	*stierlingi*	T‐West Usambaras	Ambanbugul‐32	1250	Cloud forest	89	–
*Z. senegalensis*	*stierlingi*	T‐East Usambaras	T‐East Usambaras‐33	1030	Cloud forest	66	–
		*N*				633	36
*Zosterops abyssinicus*							
*Z. abyssinicus*	*flavilateralis*	K‐Mt. Nyiru	South Horr‐34	1050	Savannah	30	30
*Z. abyssinicus*	*flavilateralis*	K‐Kitui	Nzeeu River‐35	900	Savannah	22	–
*Z. abyssinicus*	*flavilateralis*	K‐Foothills of Chyulu Hills	Umani Spring‐36	650	Savannah	33	39
*Z. abyssinicus*	*flavilateralis*	K‐Foothills of Chyulu Hills	Dembwa‐37	906	Savannah	29	–
*Z. abyssinicus*	*flavilateralis*	K‐ Foothills of Mt. Kasigau	Rukanga‐38	630	Savannah	47	–
*N*						161	61
*N* total						1223	385

### Phenotypic data

We measured five morphometric characters following De Beer et al. (2001): flattened wing length (mm), tarsus length (mm), head length (mm), body mass (g), and the eye‐ring diameter (height and width). For the latter measurements, we calculated the eye‐ring perimeter (in mm) as follows:

$$\sqrt[{2\pi }]{\frac{h^{2}+w^{2}}{2}}$$

where *h* and *w* denote the height and the width of the eye ring.

### Genetic data

Blood or feathers were preserved in pure ethanol and subsequently stored at −20°C until DNA extraction. After processing, the birds were released unharmed. DNA was extracted with the Qiagen DNeasy^™^ Blood and Tissue Kit (Hilden, Germany), following the manufacturer's protocols for tissue, blood, and feather samples (De Volo et al. 2008). PCRs were carried out in a thermal cycler (CG1‐96; Corbett Research, Qiagen, Hilden, Germany). Microsatellite loci were amplified using the Thermozym Mastermix (Molzym, Bremen, Germany, Brea, CA, USA). Fragment length analyses were performed with an automated sequencer (Beckmann Coulter, Brea, CA, USA). We genotyped the following 15 microsatellites: Cu28, Zl44, Zl41, Zl22, Zl45, Zl14, Zl54, Zl4, Zl35, Mme12, Zl18, Zl50, Zl49, Zl4, and Zl37. Further details on primer sequences and PCR conditions are given in Habel et al. (2013). All molecular data were taken from previous studies (Habel et al. 2013, 2014).

### Statistics on phenotypic and genetic data

Differences in wing length, tarsus length, head size, body mass, and eye‐ring perimeter among populations were investigated using one‐way analysis of variance, with pairwise differences tested using Tukey's HSD. Potential relationships between population mean values of these phenotypic characters were tested with linear regression models using jmp pro v. 10 (SAS Institute Inc., Cary, NC). Biometric data were integrated with genetic data using discriminant function and principal coordinates analyses (Bray–Curtis dissimilarity). Individual‐based genetic distances were calculated in genalex v. 6.4.1 (Peakall and Smouse 2012) prior to PCoA. Genotypic and phenotypic spaces (further referred to as ordinate spaces) were orthogonal and free from multi‐colinearity and were analyzed using linear discriminant analysis in R v. 3.1.2 (R Development Core Team 2014) with an equal prior setting for each population. To account for model overfitting, we randomly selected 70% of the data to train the discriminant model and repeated this 1000 times. Finally, we used general linear modeling with orthogonal sums of squares and two‐way PERMANOVA to test for correlations of genetic, morphometric, and habitat data.

Nestedness analyses were applied to assess the decline in allele incidences along a geographical gradient as suggested by Habel et al. (2012). We used the nestedness contribution (the difference in the degree of nestedness of the total allele × site matrix and a matrix where a certain site has been excluded; Saavedra et al. 2011) as measured by the NODF metric (nestedness from overlap and decreasing fill; Almeida‐Neto et al. 2008) to infer the role of each site in the decline of allele diversity. “Seriation” sorts rows and columns of a matrix of items, here allele occurrences (rows), among sites (columns) in a way to maximize the number of presences along the matrix diagonal (Leibold and Mikkelson 2002). This diagonal is equivalent to the first axis of a correspondence analysis. Ulrich and Gotelli (2013) and Ulrich et al. (2014) showed that the rank correlation *r* of row and column positions of all nonempty cells in the “seriated” matrix is a convenient measure of directional allele turnover among sites. Here, we use the respective coefficient of determination *R* ^*2*^ as the test statistic for allele turnover across our sample localities. Because raw scores of NODF and *R* ^2^ depend on matrix fill and allele numbers, we used a null model approach (Gotelli and Ulrich 2012) and compared observed NODF scores with those obtained from 1000 matrices, where occurrences within the allele × site matrix were equiprobably reshuffled. Low NODF values compared to the null expectations imply higher degrees of unexpected occurrences of alleles and therefore point to possible introgression. To test for correlation between geographical, morphological, and genetic distances, we calculated Mantel correlations between pairwise geographical (Euclidean distances), Bray–Curtis allelic and morphological dissimilarity matrices for each species.

For the microsatellite data, we tested for distortions of microsatellites through stutter bands, large allele dropout or null alleles using the program micro‐checker v. 2.0 (Van Oosterhout et al. 2004). The mean number of alleles (*A*), *AR* (allelic richness) (based on the lowest number of individuals in a population, here *N* = 7), and locus‐specific allele frequencies were calculated with fstat v. 3.1 (Goudet 1995). Percentage of observed heterozygosity (*H*_o_) and expected heterozygosity (*H*_e_), tests of HWE (Hardy–Weinberg equilibrium), and LD (linkage disequilibrium) were calculated with arlequin v. 3.5.1.3 (Excoffier et al. 2005).

Non‐hierarchical analyses of molecular variance were performed with arlequin using the microsatellite‐specific *R*‐statistics (Slatkin 1995; Selkoe and Toonen 2006). Furthermore, we calculated pairwise *D*_est_ values (Jost 2008) across all populations (excluding Eburu Forest, due to very small sampling size) with the program smogd v.1.2.5. (Crawford 2010).

We used an individual‐based Bayesian approach without a priori definition of groups, applying the program structure v. 3.1 (Hubisz et al. 2009). The batch run function was applied to carry out a total of 100 runs (10 each for one to ten clusters), that is, *K* = 1 to *K* = 10. Replicate runs allowed us to calculate mean and standard deviation for predefined K‐values. For each run, burn‐in and simulation lengths were 15,000 and 500,000, respectively. Runs were performed under the assumption of no population admixture and uncorrelated allele frequencies. As log probability values for *K* were earlier shown to be unreliable in some cases (Evanno et al. 2005), we calculated the more refined ad hoc statistic Δ*K*, based on the rate of change in the log probability of data between successive *K*‐values. In a second approach, we calculated genetic distances among populations using the program splittree (MLST databases and software – PubMLST.org) based on our microsatellite data set. We then used this genetic matrix to generate ordinary least squares and equal angle representation with the program splittree v. 4.11.3. (Huson and Bryant 2006).

## Results

### Phenotypic data

**Figure 2:**
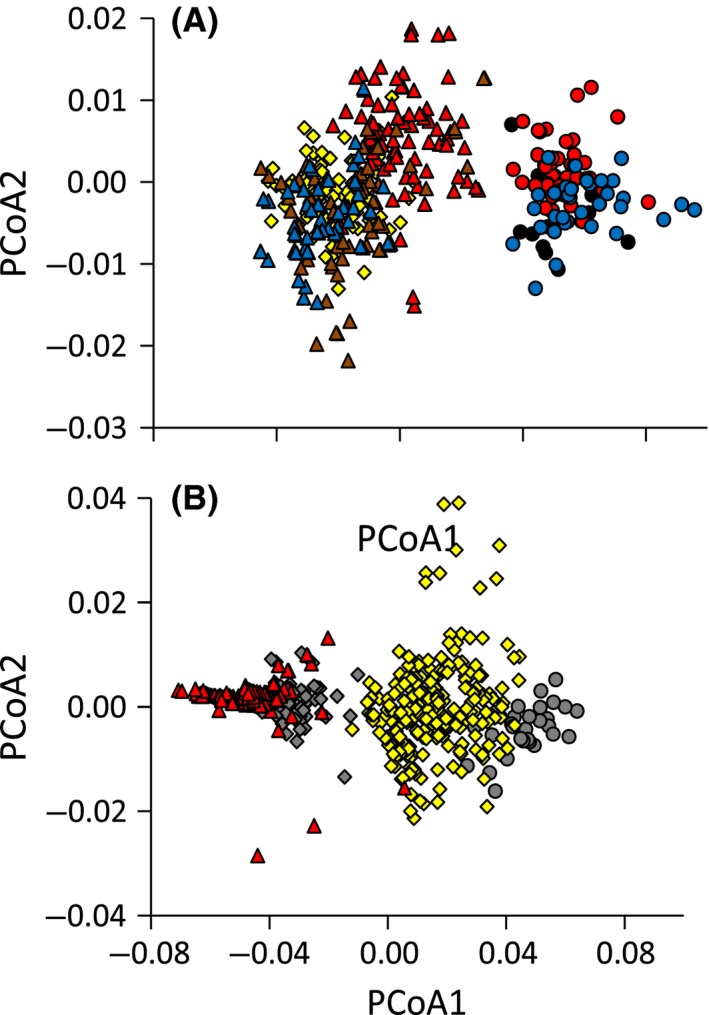
Results of principal coordinates analysis (Bray–Curtis distances) for the phenotypic data set. The first two axes explain 64.0% and 3.1% of the variance loadings in morphological variability. (A) different colors indicate the following geographical regions: red: mountain ranges of the Eastern Arc Mts. (Taita Hills, Mt. Kasigau, Mt. Meru, and Pare Mts.) and Mt. Meru; blue: Chyulu Hills; brown: central Kenyan mountain ranges with Mt. Kenya, Aberdares, Mau Escarpment; black: Kitui; and yellow: Kakamega forest. (B) northern Kenyan mountain ranges with Mt. Kulal (red), Mt. Marsabit (yellow), Mt. Nyiru (gray). Triangles: *Z. poliogaster*; rhombuses: *Z. senegalensis*; circles: *Z. abyssinicus*.

Wing length (*r* = 0.70, *P* < 0.001) and tarsus length (*r* = 0.67, *P* < 0.001) were strongly positively correlated with body weight, irrespective of species membership. Eye‐ring perimeter did not significantly depend on body weight (*r* = −0.05, *P* > 0.2), but was moderately positively correlated with head size (*r* = 0.27, *P* < 0.001). Two‐way PERMANOVA revealed significant differences in morphology among study sites (*F*_18,1166_ = 149.9, *P* < 0.001) and the three species (*F*_2,1166_ = 406.1, *P* < 0.001). Highly significant species × site interaction terms (*F*_36,1166_ = 31.35, *P* < 0.001) also pointed to intraspecific morphological differentiation. Principal coordinates analysis revealed that morphological characters separated *Z. poliogaster* and *Z. abyssinicus* (Fig. 2A and B). Some of the *Z. senegalensis* populations (Maralal, Kakamega Forest, Mt. Nyiru, and Matthews range) clustered within *Z. poliogaster,* while other *Z. senegalensis* populations were intermediate between *Z. poliogaster* and *Z. abyssinicus* (Fig. 2B). *Zosterops poliogaster* populations showed higher morphological variability compared to *Z. abyssinicus*. In particular, populations of *Z. poliogaster* from the Chyulu Hills differed strongly from those of the Eastern Arc Mountains (Fig. 2A).

**Figure 3:**
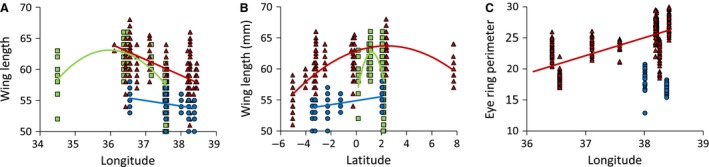
Plots of wing length (mm) (A, B) and eye‐ring perimeter (mm^2^) (C) of *Z. poliogaster* (red triangles), *Z. senegalensis* (green squares), and *Zosterops abyssinicus* (blue circles) against longitude (A, C) and latitude (B). Regressions in A: *Z. poliogaster*:* r* ^2^ = 0.34; *Z. senegalensis*:* r* ^2^ = 0.59; *Z. abyssinicus*:* r* ^2^ = 0.16. B: *Z. poliogaster*:* r* ^2^ = 0.60; *Z. senegalensis*:* r* ^2^ = 0.30; *Z. abyssinicus*:* r* ^2^ = 0.21., and C: *r* ^2^ = 0.47. All *P* < 0.0001. For *Z. senegalensis,* no eye‐ring data were available.

Regressions of wing length (Fig. 3A and B) and eye‐ring perimeter (Fig. 3C) with longitude and latitude revealed geographical trends in *Z. poliogaster*,* Z. senegalensis,* and *Z. abyssinicus*. Wing length of *Z. poliogaster* and *Z. senegalensis* peaked at intermediate longitude and latitude (Fig. 3A and B). Eye‐ring perimeters of *Z. poliogaster*, but not *Z. abyssinicus*, increased from northwest to southeast (Fig. 3C). The high intrapopulation variability in morphological characters caused an insignificant Mantel correlation (*r* = −0.09, permutation *P* > 0.5) between morphological Bray–Curtis and geographical Euclidean distances. Finally, we observed a significant increase in wing length (*r* = 0.93, *P* < 0.001) and body weight (*r* = 0.89, *P* < 0.001) with the altitude, while eye‐ring perimeter was not significantly related to altitude (*r* = 0.47, *P* > 0.1). Morphometric raw data are provided in Appendix S1.

### Genetic data

Effects from null alleles and large allele dropout were detected for the loci Zl44, Zl54, Zl35, and Mme12. No linkage disequilibrium was detected between any pair of loci after Bonferroni correction. Deviations from HWE were detected for loci Cu28, ZL41, and ZL45. As only few loci and populations were out of equilibrium, all data were included in subsequent analyses.

The number of alleles per locus ranged between 4 (*Mme12*,* Zl54*) and 17 (*Zl22*) alleles, with a mean of 8.9 (±4.5 SD). Among the 133 alleles across all loci, 38 were restricted to single mountain massifs or populations. Allele frequencies for all loci and populations are given as Appendix S2. Genetic diversity (average number of *A* (alleles), *AR*, and percentage of expected *H*_e_ (heterozygosity) and observed *H*_o_ (heterozygosity) showed higher values for the two populations analyzed for *Z. abyssinicus* in comparison to populations assigned to *Z. poliogaster* (*t*‐test, *P* < 0.001); however, no further significant differences among the other taxa were detected. Values of Genetic diversity measures and mean values of morphological characters are given in Appendix S3.

**Figure 4:**
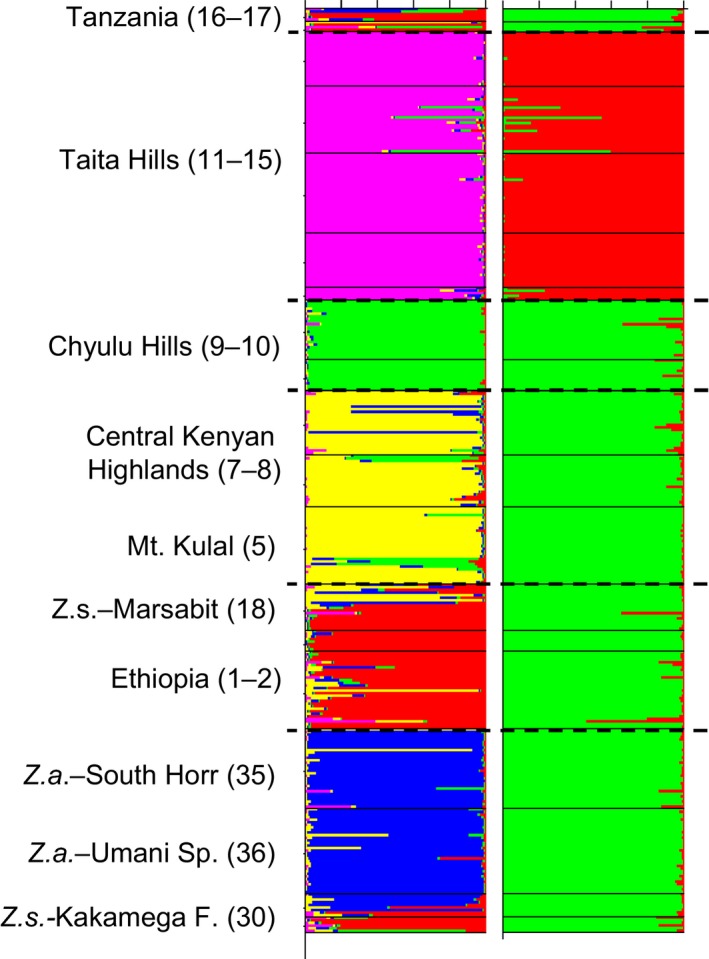
Bayesian structure analyses calculated with the program structure (Hubisz et al. 2009) for all species and populations analyzed testing *K*‐values from 1 to 10. Results supported by highest ∆*K*‐values for *K* = 2 (right plot) and *K* = 5 (left plot) are presented. Names of mountain ranges and the respective number of locations coincide with Table 1.

Structure provided the highest support for *K* = 2 (mean probability values and respective Δ*K*‐values are given in Appendix S4), dividing the populations from the Taita Hills from all other populations analyzed (Fig. 4). However, as Hausdorf and Hennig (2010) suggest that clustering in only two groups may sometimes prove misleading results, we also visualized the second best *K*‐value suggesting five groups (*K* = 5). Under this model, the populations were divided into the following five clusters: (1) populations from the Taita Hills together with Mt. Kasigau, (2) the Chyulu Hills, (3) all locations from the central Kenyan highlands including Mt. Kulal, (4) the lowland species *Z. abyssinicus*, clustering together with the *Z. senegalensis* population from Kakamega Forest, and (5) populations from the Ethiopian Highlands with individuals of *Z. senegalensis* sampled at Mt. Marsabit. The populations of *Z. poliogaster* from Tanzania (Pare Mts., Mt. Meru) were genetically not clearly assigned to any specific cluster (remark that these populations are represented by only few individuals) (Fig. 4). The genetic clusters obtained from Splittree show identical assignments of the individuals, with two main groups (individuals from the Taita Hills including Mt. Kasigau versus all other populations). Individuals from the three taxa (*Z. poliogaster*,* Z. senegalensis,* and *Z. abyssinicus*) strongly intermix and do not form distinct branches. Results are given in Appendix S5.

AMOVAs (Analyses of molecular variance) suggested strong genetic differentiation among all species and populations explaining 78.8% of the molecular variance (*R*_ST_ = 0.3694, *P* < 0.0001). The intraspecific differentiation was highest within *Z. poliogaster* explaining 69.6% of the variance (*R*_ST_ = 0.3572, *P* < 0.0001) and lowest for *Z. senegalensis* (Marsabit vs. Kakamega Forest, 39.6% (*R*_ST_ = 0.1372, *P* < 0.01). We found no significant differentiation between the populations of *Z. abyssinicus* (−1.3293, *R*_ST_ = 0.0085, nsec). *D*_est_ values ranged between 0.001 and 0.315, with highest divergence between the populations from Ethiopia and Mt. Kasigau. Pairwise *D*_est_ values are given in Table 2.

**Table 2:** Pairwise *D*_est_ values between all pairs of populations (except Eburu forest) calculated with the program smogd. White cells = *Zosterops poliogaster*; Light gray cells = *Z. senegalensis*; Dark gray cells = *Z. abyssinicus*. Numbers of locations coincide with numbers given in Table 1

	1	2	5	7	8	9	10	11	12	13	14	15	16	17	18	30	34	36
1	–	–	–	–	–	–	–	–	–	–	–	–	–	–	–	–	–	–
2	0.033	–	–	–	–	–	–	–	–	–	–	–	–	–	–	–	–	–
5	0.114	0.083	–	–	–	–	–	–	–	–	–	–	–	–	–	–	–	–
7	0.122	0.056	0.051	–	–	–	–	–	–	–	–	–	–	–	–	–	–	–
8	0.091	0.048	0.044	0.000	–	–	–	–	–	–	–	–	–	–	–	–	–	–
9	0.171	0.094	0.086	0.069	0.063	–	–	–	–	–	–	–	–	–	–	–	–	–
10	0.161	0.101	0.070	0.071	0.060	0.006	–	–	–	–	–	–	–	–	–	–	–	–
11	0.259	0.148	0.155	0.100	0.102	0.148	0.153	–	–	–	–	–	–	–	–	–	–	–
12	0.210	0.106	0.174	0.100	0.095	0.147	0.165	0.008	–	–	–	–	–	–	–	–	–	–
13	0.250	0.142	0.146	0.095	0.096	0.135	0.142	0.000	0.008	–	–	–	–	–	–	–	–	–
14	0.244	0.125	0.143	0.089	0.095	0.138	0.145	0.000	0.010	0.001	–	–	–	–	–	–	–	–
15	0.315	0.186	0.179	0.127	0.135	0.183	0.181	0.013	0.040	0.015	0.024	–	–	–	–	–	–	–
16	0.052	0.037	0.066	0.039	0.026	0.067	0.098	0.096	0.066	0.081	0.077	0.146	–	–	–	–	–	–
17	0.077	0.077	0.138	0.088	0.062	0.091	0.115	0.098	0.100	0.093	0.078	0.113	0.012	–	–	–	–	–
18	0.132	0.063	0.062	0.051	0.046	0.078	0.087	0.118	0.109	0.108	0.089	0.148	0.103	0.111	–	–	–	–
30	0.128	0.071	0.052	0.054	0.062	0.082	0.091	0.127	0.133	0.112	0.094	0.175	0.096	0.117	0.007	–	–	–
34	0.050	0.053	0.070	0.069	0.061	0.112	0.099	0.131	0.134	0.120	0.099	0.163	0.026	0.043	0.068	0.065	–	–
36	0.088	0.061	0.059	0.048	0.040	0.085	0.109	0.113	0.116	0.102	0.080	0.171	0.081	0.096	0.020	0.023	0.069	–

Allele occurrences among sites were significantly nested (observed NODF = 53.7, expected NODF: 33.7 ± 0.7, *P* < 0.0001). This ordered loss of alleles occurred along a gradient from northwest to southeast (*r* = 0.58, *P* < 0.01). Only the Pare Mts., Mt. Kasigau, Bura (Taita Hills), and Simba Valley (Chyulu Hills) differed from this nested appearance having negative nestedness contributions and an idiosyncratic number of allele occurrences. Accordingly, allele turnover among sites was lower than expected by chance (observed *R* ^2^ = 0.13, expected *R* ^2^ = 0.17).

### Combining phenotypic and genetic data

The distribution of alleles among sites was not significantly linked with the distribution of morphological features. A Bray–Curtis dissimilarity based Mantel test did not return a significant correlation between morphological dissimilarity (body weight, wing and tarsus length, and head length) of the populations and their respective genetic dissimilarity (all *r* < 0.2; *P* > 0.1). Nevertheless, the morphological and genetic analyses suggested strong differentiation among the study regions (Figs. 2, 4). The intraspecific genetic and morphological differentiation among the *Z. poliogaster* populations was as high as the interspecific differentiation between species.

## Discussion

Our data showed strong phenotypic and genetic splits among the three taxa, but even stronger splits among local populations within single species. Specifically, strong intraspecific differentiation was found between isolated mountain populations of *Z. poliogaster*. Shallower splits were detected among the *Z. abyssinicus* and *Z. senegalensis* populations, both thought to represent a comparatively panmictic distribution. We found significant spatial patterns of phenotypic variation with longitudinal, latitudinal, and altitudinal gradients in all three species. However, morphological and genetic traits showed no congruent signature as expected as we suggested that different traits (morphology and genetics) evolve under the regime of different evolutionary drivers. In the following, we will discuss our findings against the background of potential effects from different distribution settings (disjunction versus panmixis) and contrasting evolutionary drivers (natural and sexual selection, and drift); finally, we make some taxonomic recommendations for this bird species complex.

### Effects from disjunction and panmixia

Intraspecific divergence was more pronounced than the divergence between the currently recognized species. Our analyses revealed mountain‐specific genetic and phenotypic clusters for *Z. poliogaster*, with a main split found between populations from the Eastern Arc Mountains (i.e., the Taita Hills with Mt. Kasigau) and all other studied populations. We further detected shallower splits among the other mountain populations in line with previous studies (cf. Habel et al. 2013; Cox et al. 2014); similarly, relatively low levels of intraspecific divergence were found in the two lowland species (Melo et al. 2011; Cox et al. 2014). These populations have idiosyncratic allele distributions as revealed by the nestedness analysis and formed distinct genetic and phenotypic clusters. Various studies on other organisms explain such strong genetic differentiation by long‐lasting geographical isolation of mountain ranges providing long time spans for independent evolutionary processes (cf. White 1978; Lovett and Wasser 1993). Strong within‐taxon differentiation for both genetic and phenotypic traits has already been identified for many species in East African mountain ranges (e.g., the Eastern Arc Mts., Fjeldså and Lovett 1997; Kebede et al. 2007; Tolley et al. 2011). Relatively long geological time spans (going back to Miocene/Oliogcene) of distinct evolutionary trajectories in allopatry are assumed to be the main drivers of differentiation of these tropical forest species (Tolley et al. 2011; Measey and Tolley 2011; but see also Cox et al. 2014). This has also been shown for various animal species (Kahindo et al. 2007), as for example for the bird species Tiny Greenbul *Phyllastrephus debilis* along an altitudinal gradient in the Eastern Arc Mts. (Fuchs et al. 2011). Fuchs and colleagues found that the two distinct subspecies are the product of long‐term isolation and their contact zone is the result of a recent secondary contact rather than a recent divergence via disruptive selection across altitudinal gradients.

Similar to the examples in the previous paragraph, the panmicticly distributed species *Z. senegalensis* and *Z. abyssinicus* show high levels of genetic and phenotypic substructuring (see Cox et al. 2014). This is rather surprising as local populations of these species are thought to be connected by high levels of gene flow in the relatively homogenous habitat matrix of the lowland savannahs. Hence, the differentiation may be the result either of past barriers to gene flow or intrinsic barriers, such as behavioral isolation which may have occurred gradually and became strong enough at the edges of the distribution, similar to what is seen in ring species (e.g., Irwin 2000; Irwin et al. 2005).

Results from our microsatellite analyses support previous findings based on mitochondrial sequences and AFLP data (Cox et al. 2014), providing strong evidence for the polyphyly of *Z. poliogaster* and the other East African species of this genus. As such, white‐eye populations on distinct mountains do not represent a single species that found itself in a fragmented range, but more likely represent multiple instances of independent adaptation and subsequent speciation of lowland populations extending into high elevation areas (cf. Cox et al. 2014). Once there, they independently diverged into “high elevation phenotypes” that share some characteristics (Husemann et al. 2012). Alternatively, the phenotypic similarities may be the result of morphological conservatism, a phenomenon predicting that morphological traits remain stable as a result of similar selective pressures despite strong genetic divergence (e.g., Austin 1995; Lavoué et al. 2011). From our data, it is difficult to distinguish between the two processes. However, independent of which process has led to these similar morphologies, the high resemblance of mountain populations has led taxonomists using external morphological characteristics, notably plumage to lump these populations within the same species.

### Effects from natural and sexual selection, and drift

Phenotypic and genetic variation did not show a congruent pattern of spatial distribution. Our nestedness analysis pointed to latitudinal and longitudinal, as well as altitudinal relationships for wing length and eye‐ring perimeter. As habitat type gradually changes with altitude (moist and cool cloud forest at higher elevation versus dry and hot lowland savannah) our results also suggest differences in morphological structure among habitat types. Correlations between altitude and the expression of morphological characteristics in white‐eye bird species have been previously detected (Moreau 1957). Such clinal changes have been attributed to gradual variation in ecological conditions and the resulting selective environment (Storz 2002). However, such geographical clines in morphological traits have been interpreted differently, depending on the type of character (shape of the bill = usage of specific resources; plumage coloration = sexual selection, Irwin 2000; Irwin et al. 2005). If traits are under sexual selection, clinal geographical variation may facilitate reproductive isolation, which can ultimately lead to speciation (Lande 1982).

Our data suggest that the divergence of the studied phenotypic traits may be mainly driven by selection and not stochastic processes, which drive the divergence of neutral traits, in our case microsatellites (Leinonen et al. 2006, 2008, 2013; Sæther et al. 2007). Phenotypic characters are known to be under strong environmental (and sexual) selection in birds (Endler 1982; Hendry et al. 2000; Smith et al. 2001). Thus, our results suggest that similarities in morphology may most probably be the result of convergent evolution in combination with morphological conservatism in response to similar ecological pressures. The importance of eco‐geographical phenotypic variants is further underlined by similar findings in several other white‐eye groups (cf. Moreau 1957; Milá et al. 2010; Melo et al. 2011; Oatley et al. 2011, 2012), which likewise showed that morphological characters such as tarsus and head length and body mass are strongly correlated with the distribution of ecosystems. These small‐scale differentiation processes may possibly explain some of the past taxonomic confusion in this genus.

### Toward a new taxonomic arrangement

The taxonomic status of many populations of the genus *Zosterops* is under debate. While the genus is generally recognized as a “great speciator” (Moyle et al. 2009; Melo et al. 2011), comparatively few species are described from East Africa. This is partially due to the lack of good phenotypic characters distinguishing the species and the high variation that is found within populations (Zimmerman et al. 1996; Fry et al. 2000). However, after a series of genetic and phenotypic studies (Habel et al. 2013, 2014; Cox et al. 2014; Husemann et al. 2014b, 2015b), we now have strong evidence for the polyphyly of most species in this species complex, with a relatively clear differentiation into distinct taxa, which likely evolved in the wake of colonizations of isolated mountain blocks (vast geographical distances in combination with behavioral flightlessness) and subsequent independent adaptation to local highland conditions (Cox et al. 2014).

Based on our data, we suggest to divide *Z. poliogaster* into at least three distinct species, which have partially been suggested as valid species in the past: (1) *Zosterops silvanus* including the populations from the Taita Hills and Mt. Kasigau, supported by mtDNA sequences (Cox et al. 2014) and a high proportion of private alleles in microsatellites (this contribution), as well as the phenotypic and bioacoustic data presented here and in Husemann et al. (2014b), (2) *Zosterops mbuluensis* from Chyulu Hills, supported by mtDNA sequences (Cox et al. 2014), microsatellites, and phenotypic data presented here and in previous studies, and (3) *Z. kikuyuensis* from the central Kenyan highlands (i.e., the Aberdares and Mt. Kenya) supported by mtDNA and microsatellite data, but also by morphological data (this contribution, also see Cox et al. 2014). This taxonomic treatment was already suggested by Collar et al. (1994) for *Z. silvanus*, and for *Z. mbuluensis* and *Z. kikuyuensis* by Zimmerman et al. (1996), and was supported by the results of Cox et al. (2014). However, the taxonomic re‐assignment suggested here is based on the phylogenetic species concept (De Queiroz 2007), and further evidence is necessary to prove this taxonomic split according the classical biological species concept. The status of *Z. senegalensis*, of which some populations show strong phenotypic and genetic similarities (microsatellites this study, mtDNA Cox et al. 2014) with *Z. poliogaster,* remains questionable and requires critical evaluation by including additional populations.

## Conflict of Interest

None declared.

## Data Accessibility

All data used in this manuscript is provided as online supplemental material S1–S3.

## Supporting information


**Appendix S1.** Raw data of morphological measurements; Wing length (mm), tarsus length (mm), body weight (g) and eye ring length and height (mm).

**Appendix S2.** Allele frequencies of all populations and microsatellite loci analysed. Site numbers coincide with other supplementary material, figures and tables of the article.

**Appendix S3.** Summary data of sample sites, with species, subspecies, exact geographical location, altitude, and mean values of all genetic and morphological characters analysed: Total number of alleles (A), allelic richness (AR), number of private alleles restricted to single mountain massifs (AP), percentage of expected heterozygosity (He) and observed heterozygosity (Ho). Site numbers match with those of Fig. 1.

**Appendix S4.** Mean probability values with standard deviations calculated based on ten runs each for *K* = 1–10, and respective ∆*K*‐values.

**Appendix S5.** Neighbornet generated with the program Splitstree.
