# Supplementary material for: Evolution along the Great Rift Valley: phenotypic and genetic differentiation of East African white‐eyes (Aves, Zosteropidae)
Source: Ecol Evol. 2015 Oct 12;5(21):4849–62. doi: 10.1002/ece3.1735 (PMC4662327; doi:10.1002/ece3.1735)
Supplement: Supplementary file 4 — Appendix S4. Mean probability values with standard deviations calculated based on ten runs each for K = 1–10, and respective ∆K‐values. [file ECE3-5-4849-s004.doc]

Supplementary Material Electronic Appendix S4: Mean probability values with standard deviations calculated based on ten runs each for K=1-10, and respective ∆K values.

| **K** | **1** | **2** | **3** | **4** | **5** | **6** | **7** | **8** | **9** | **10** |
| --- | --- | --- | --- | --- | --- | --- | --- | --- | --- | --- |
| **1** | -9827.4 | -8452.9 | -8024.0 | -7568.2 | -7255.6 | -7062.4 | -6940.0 | -6892.7 | -6865.6 | -6828.2 |
| **2** | -9827.4 | -8453.2 | -8024.7 | -7598.9 | -7256.4 | -7059.4 | -6933.1 | -6908.8 | -6878.5 | -6837.1 |
| **3** | -9827.5 | -8452.3 | -8025.3 | -7600.3 | -7256.0 | -7062.0 | -6936.7 | -6893.0 | -6864.4 | -6850.5 |
| **4** | -9827.5 | -8453.5 | -8025.8 | -7583.3 | -7257.0 | -7161.6 | -6939.5 | -6898.0 | -6897.9 | -6829.6 |
| **5** | -9827.4 | -8453.7 | -7937.2 | -7572.4 | -7255.4 | -7131.7 | -6936.9 | -6887.3 | -6872.6 | -6949.1 |
| **6** | -9827.5 | -8453.3 | -7936.1 | -7598.0 | -7257.4 | -7061.4 | -6935.6 | -6893.1 | -6858.8 | -6860.7 |
| **7** | -9827.5 | -8452.8 | -8026.2 | -7630.1 | -7257.2 | -7063.5 | -6936.4 | -6907.4 | -6860.1 | -6860.7 |
| **8** | -9827.4 | -8452.8 | -7936.6 | -7599.4 | -7258.6 | -7061.6 | -6939.5 | -6888.8 | -6874.0 | -6902.3 |
| **9** | -9827.4 | -8454.0 | -7937.6 | -7597.6 | -7255.7 | -7060.2 | -6936.4 | -6897.8 | -6878.2 | -6859.5 |
| **10** | -9827.4 | -8452.8 | -7937.1 | -7598.9 | -7257.1 | -7061.7 | -6935.5 | -6897.8 | -6859.4 | -6852.7 |
| **Mean ±SD** | **9827.44 ±0.05** | **8453.13 ±0.50** | **7981.06 ±46.53** | **7594.71 ±17.29** | **7256.64 ±1.00** | **7078.55 ±36.59** | **6936.96 ±2.15** | **6896.47 ±7.13** | **6870.95 ±12.10** | **6863.04 ±36.87** |
| **∆K** | **-** | **1776.67** | **1.84** | **2.79** | **159.59** | **0.99** | **46.95** | **2.09** | **1.46** | **-** |
