# Supplementary figures and images for: Evolution along the Great Rift Valley: phenotypic and genetic differentiation of East African white‐eyes (Aves, Zosteropidae)
Source: Ecol Evol. 2015 Oct 12;5(21):4849–62. doi: 10.1002/ece3.1735 (PMC4662327; doi:10.1002/ece3.1735)

## Slide 1
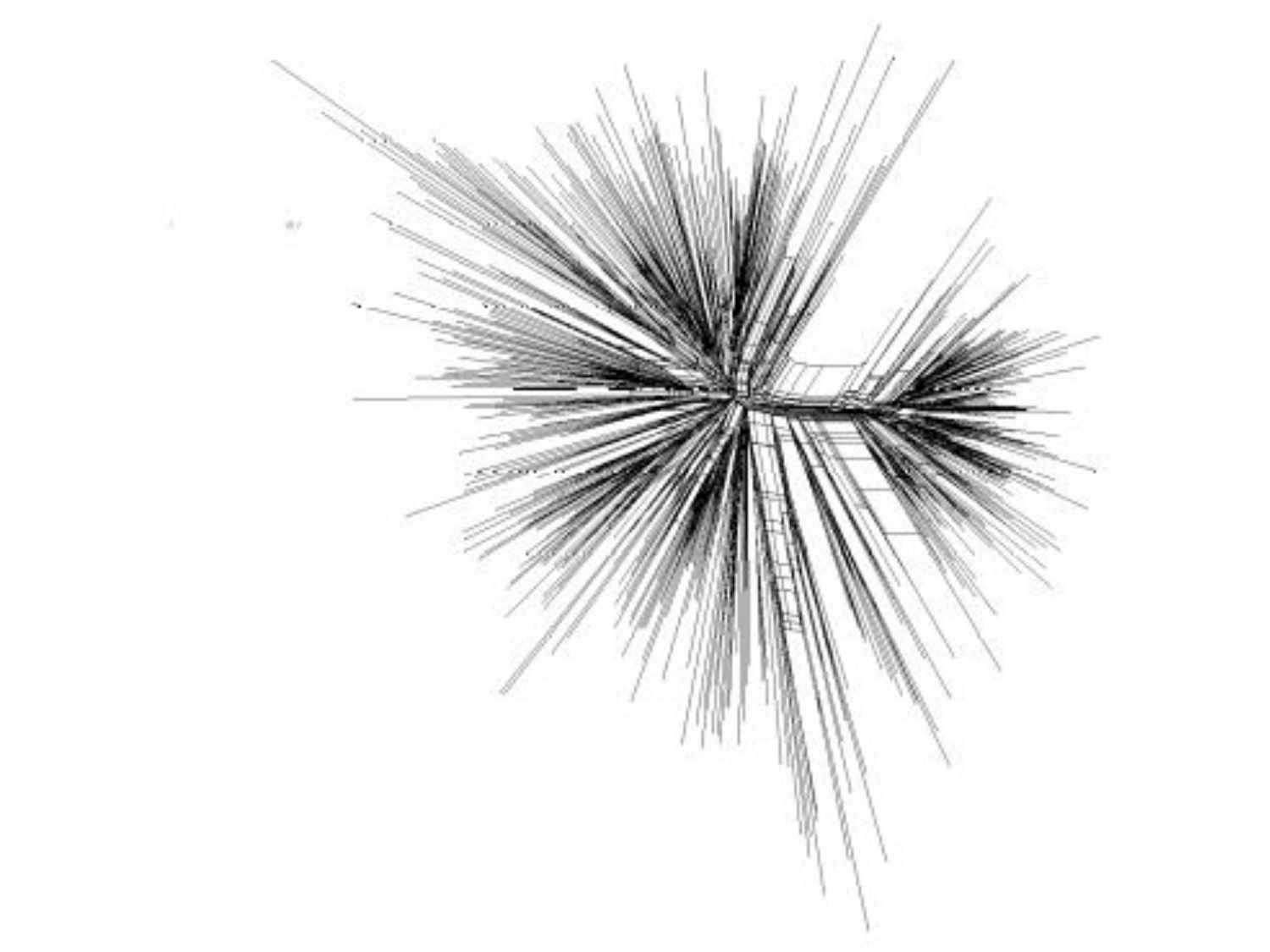

#

## Slide 2
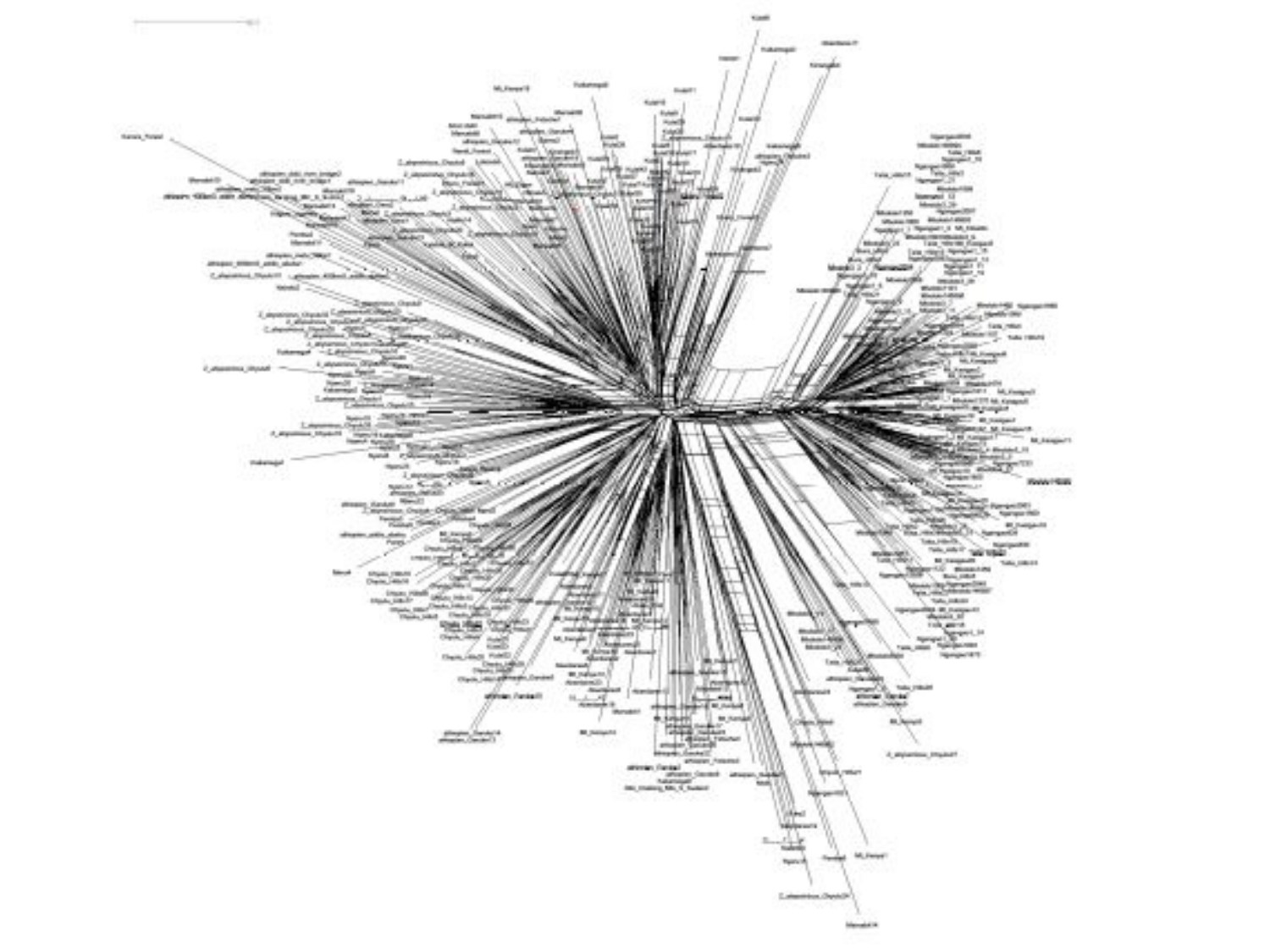

Supplement: Supplementary file 5 — Appendix S5. Neighbornet generated with the program Splitstree. [file ECE3-5-4849-s005.ppt]
